# Supplementary material for: Potential Source and Transmission Pathway of Gut Bacteria in the Diamondback Moth, Plutella xylostella
Source: Insects. 2023 May 31;14(6):504. doi: 10.3390/insects14060504 (PMC10299291; doi:10.3390/insects14060504)
Supplement: Supplementary file 1 [file insects-14-00504-s001.zip › insects-2282385-supplementary.pdf]

Supplementary Information

Potential source and transmission pathway of gut bacteria in the diamondback moth, *Plutella xylostella*

Shuncai Han[1,2,3†], Qianqian Ai[1,2,3†], Xiaofeng Xia[1,2,3*]

[1]State Key Laboratory of Ecological Pest Control for Fujian and Taiwan Crops, Fujian Agriculture and Forestry University, Fuzhou 350002, China;

[2]Institute of Applied Ecology, Fujian Agriculture and Forestry University, Fuzhou 350002, China; [3]Key Laboratory of Integrated Pest Management for Fujian-Taiwan Crops, Ministry of Agriculture and rural affairs, Fuzhou 350002, China.

Running Head: Transmission of DBM gut microbiota

† These authors contributed equally to this work.

*Correspondence to X.X. (Email: xiaofengxia@fafu.edu.cn)

This file includes:

Supplementary Figures 1-5

Supplementary Tables 1-10

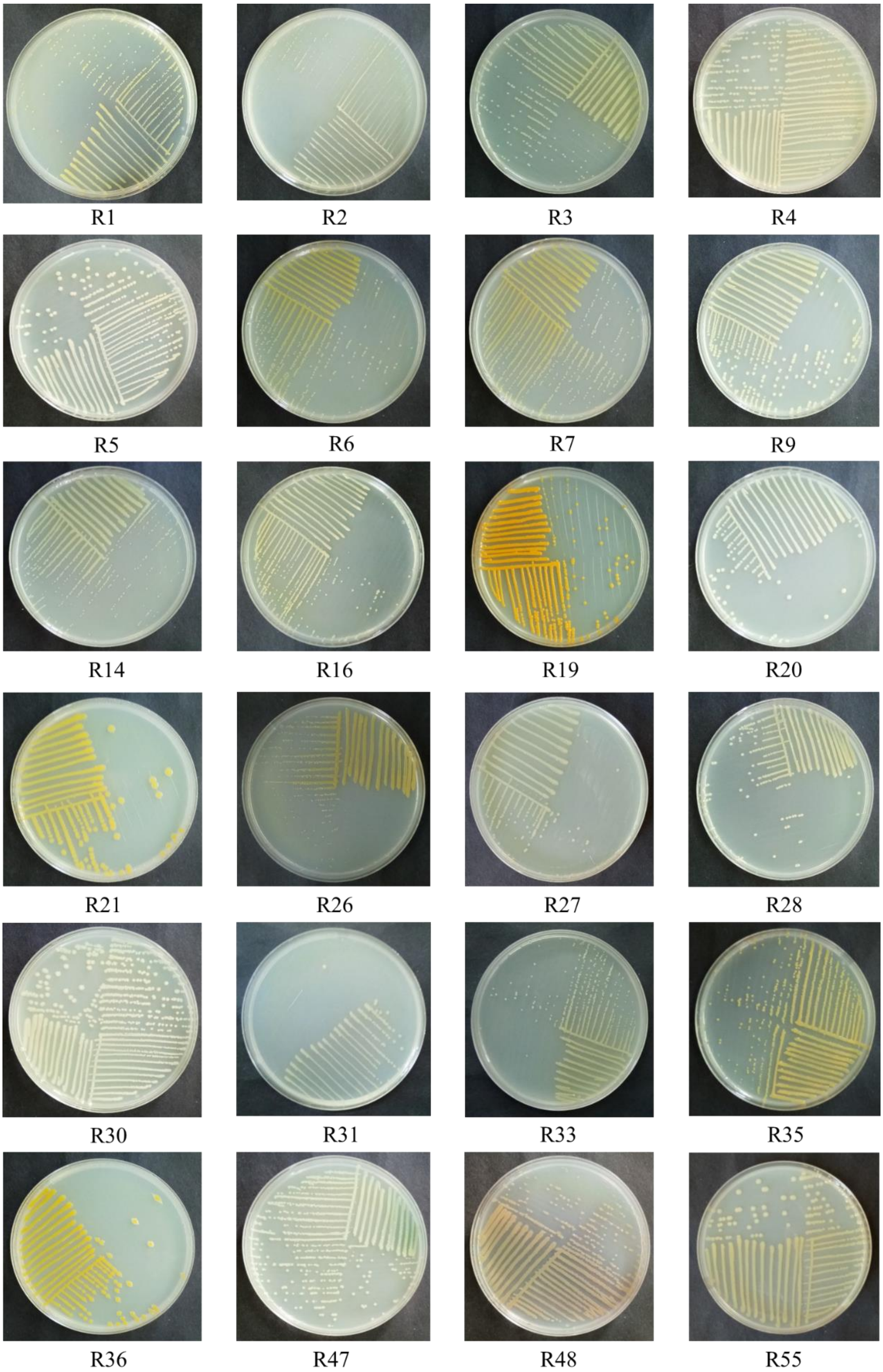

Figure S1. Bacteria strains isolated and purified from radish sprouts.

R: radish sprouts

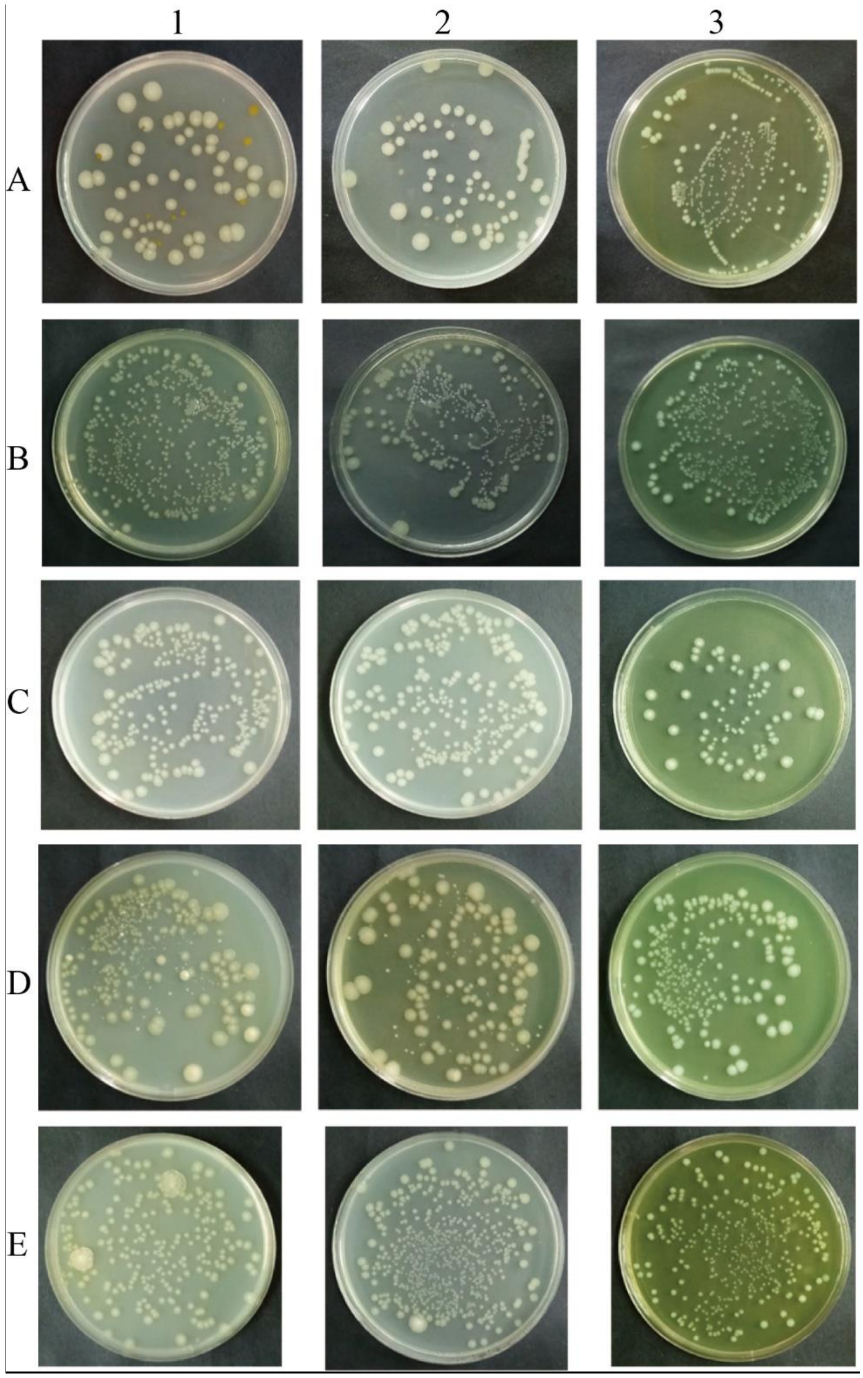

Figure S2. Isolation of bacteria from *P. xylostella* at different stages.
The vertical columns of 1, 2 and 3 represent Luria-Bertani medium, Nutrient Agar medium and Anaerobic Agar medium respectively; The horizontal columns of A, B, C, D and E represent the 4th instar larval gut, pupal gut, adult gut, ovaries and eggs of *P. xylostella*.

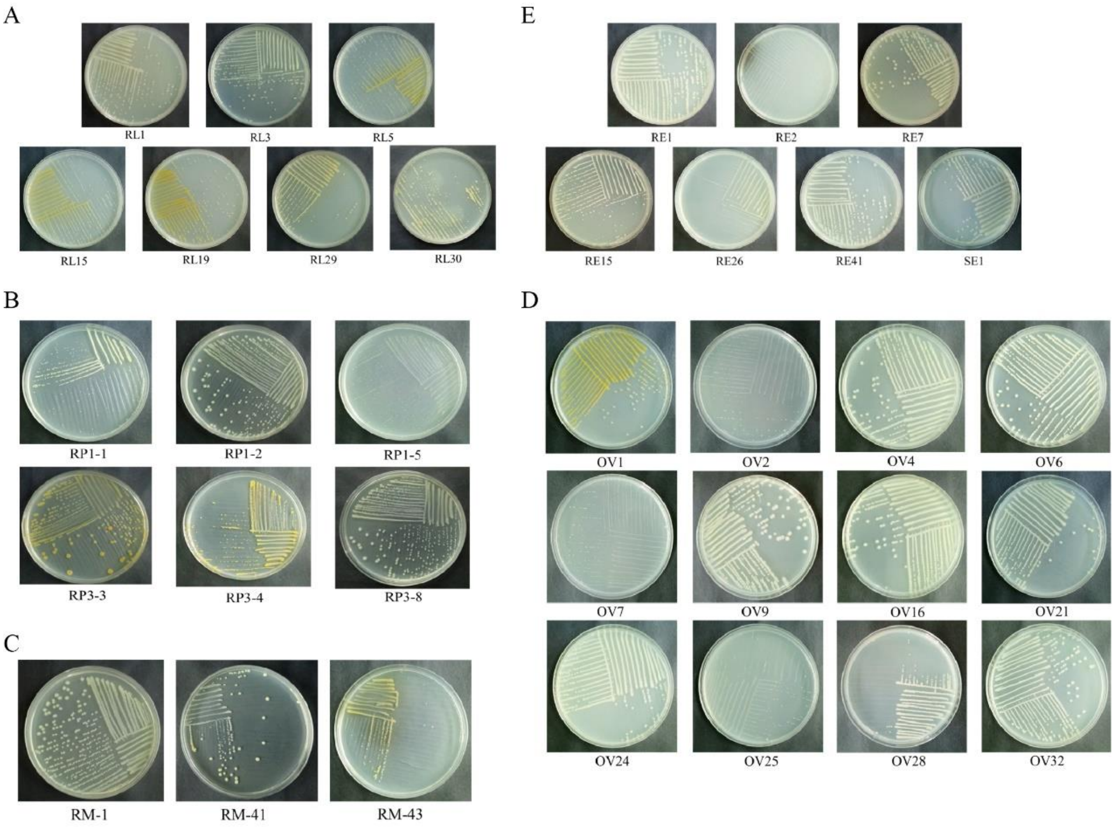

Figure S3. Bacteria strains isolated and purified from *P. xylostella* at different stages. A, B, C, D and E represent the 4th instar larval gut, pupal gut, adult gut, ovaries and eggs of *P. xylostella*. RL represents gut bacteria in the 4th instar larvae of *P. xylostella*, RP represents gut bacteria in the pupae of *P. xylostella*, RM represents gut bacteria in adult *P. xylostella*, OV represents ovarian bacteria in *P. xylostella*, and SE and RE represent bacteria in eggs of *P. xylostella.*

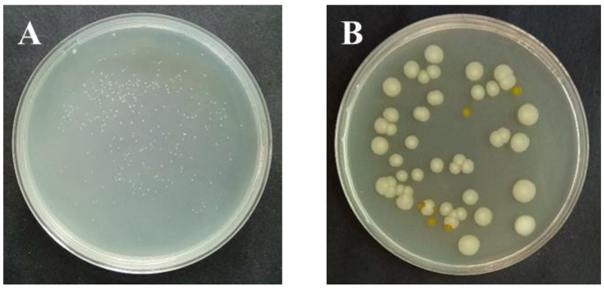

Figure S4. Isolation of gut bacteria from 4th instar larvae of *P. xylostella*
A: Gut of 4th instar larvae feeding on artificial diet; B: Gut of 4th instar larvae feeding on radish sprout

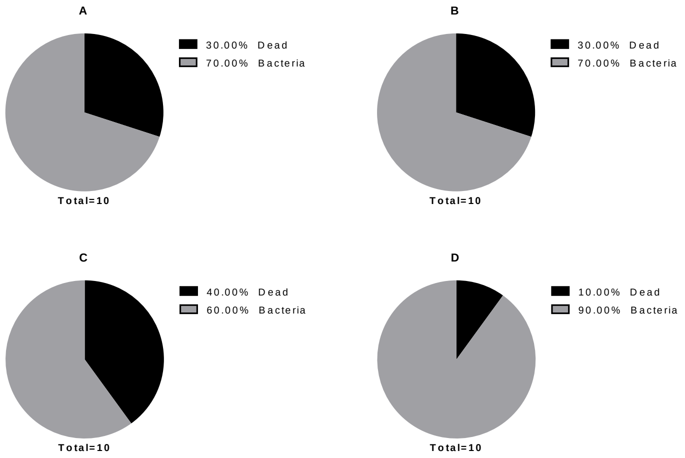

Figure S5. Statistics on the number of *P. xylostella* with *Enterobacter* spp. RE1-KN in mixed feeding

A: The first repetition group, B: The second repetition group, C: The third repetition group, D: The fourth repetition group. The black part represents the number of dead *P. xylostella* and the gray part represents the number of *P. xylostella* with *Enterobacter* spp. RE1-KN.

Table S1 The amplification system for PCR

Name	Dosage
2×Phanta Max Buffer	12.5 μL
Template DNA	1 μL
T7	1 μL
T7-ter	1 μL
dNTP Mix	0.5 μL
Phanta Max Surper-Fidelity DNA Polymerase	0.5 μL
ddH_2O	Add to 25 μL

Table S2 The response procedure for PCR

Procedure	Temperature	Time	Cycle
Pre-denaturation	95℃	3 min	1
Denaturation	94℃	30 s	30
Annealing	58℃	30 s	
Extension	72℃	1 min	
Thorough extension	72℃	10 min	1

Table S3 Blast-based alignment of 16S rDNA of bacteria from radish sprouts

Clone No.	Accession Number	Blast sequences with highest identity（Accession Number）（%）
R1	MH141447	*Curtobacterium* sp.（KR906484.1）（100%）
R2	MH141448	*Arthrobacter* sp.（EU086821.1）（99%）
R3	MH141449	*Leifsonia* sp.（FJ006907.1）（100%）
R4	MH141450	*Leclercia adecarboxylata*（LC170015.1）（100%）
R5	MH141451	*Enterobacter* sp.（KY979136.1）（100%）
R6	MH141452	*Pantoea vagans*（KP099965.1）（100%）
R7	MH141453	*Pantoea agglomerans*（KJ210675.1）（99%）
R9	MH141454	*Salmonella* sp.（HM365947.1）（99%）
R14	MH141455	*Stenotrophomonas maltophilia*（KY606632.1）（100%）
R16	MH141456	*Burkholderia* sp.（KU736928.1）（100%）
R19	MH141457	*Chryseobacterium* sp.（KU360134.1）（99%）
R20	MH141458	*Pseudomonas fulva*（KY511074.1）（100%）
R21	MH141459	*Erwinia* sp.（KR189833.1）（99%）
R26	MH141460	*Dyella jiangningensis*（NR 121724.1）（100%）
R27	MH141461	*Delftia* sp.（MG650648.1）（100%）
R28	MH141462	*Kosakonia cowanii*（KF411346.1）（99%）
R30	MH141463	*Enterobacter asburiae*（KY938112.1）（100%）
R31	MH141464	*Pandoraea pnomenusa*（KU252675.1）（100%）
R33	MH141465	*Ralstonia mannitolilytica*（MG571736.1）（100%）
R35	MH141466	*Chitinophaga* sp.（KC119172.1）（98%）
R36	MH141467	*Pantoea* sp.（MF962590.1）（100%）
R47	MH141468	*Cedecea* sp.（KY780238.1）（100%）
R48	MH141469	*Pseudomonas aeruginosa*（MG214521.1）（100%）
R55	MH141470	*Cronobacter sakazakii*（GU227677.1）（100%）

Note: R indicates that bacteria of the radish sprouts.

Table S4 Blast-based alignment of 16S rDNA from the 4th larval gut bacteria of *P. xylostella*

Clone No.	Accession Number	Blast sequences with highest identity（Accession Number）（%）
RL1	MH141471	*Enterobacter ludwigii*（MF101037.1）（100%）
RL3	MH141472	*Cedecea* sp.（KY780238.1）（100%）
RL5	MH141473	*Pantoea agglomerans*（KF465834.1）（100%）
RL15	MH141474	*Pantoea* sp.（MF962590.1）（100%）
RL19	MH141475	*Pantoea vagans*（KC139414.1）（99%）
RL29	MH141476	*Corynebacterium pollutisoli*（NR_151947.1）（100%）
RL30	MH141477	*Corynebacterium xerosis*（KU252662.1）（100%）

Note: RL indicates that bacteria of the 4th larval gut.

Table S5 Blast-based alignment of 16S rDNA from the pupal gut bacteria of *P. xylostella*

Clone No.	Accession Number	Blast sequences with highest identity（Accession Number）（%）
RP1-1	MH141481	*Kocuria palustris*（JQ085396.1）（100%）
RP1-2	MH141482	*Enterobacter ludwigii*（MF101037.1）（100%）
RP1-5	MH141483	*Enterococcus* sp.（KC243406.1）（99%）
RP3-3	MH141484	*Microbacterium arborescens*（KU933479.1）（100%）
RP3-4	MH141485	*Brachybacterium nesterenkovii*（KP240956.1）（99%）
RP3-8	MH141486	*Enterobacter* sp.（KR189383.1）（99%）

Note: RP indicates that bacteria of the pupal gut.

Table S6 Blast-based alignment of 16S rDNA from the adult gut bacteria of *P. xylostella*

Clone No.	Accession Number	Blast sequences with highest identity（Accession Number）（%）
RM-1	MH141487	*Enterobacter ludwigii*（MF101037.1）（100%）
RM-41	MH141488	*Brachybacterium* sp.（KU560280.1）（99%）
RM-43	MH141489	*Pseudomonas fulva*（NR104280.1）（100%）

Note: RM indicates that bacteria of the adult gut.

Table S7 Blast-based alignment of 16S rDNA from the adult ovary bacteria of *P. xylostella*

Clone No.	Accession Number	Blast sequences with highest identity（Accession Number）（%）
OV1	MH137691	*Pantoea agglomerans*（KY127366.1）（99%）
OV2	MH137692	*Carnobacterium maltaromaticum*（MF988693.1）（100%）
OV4	MH137693	*Enterobacter cancerogenus*（NR044977.1）（99%）
OV6	MH137694	*Raoultella ornithinolytica*（NR044799.1）（100%）
OV7	MH137695	*Carnobacterium gallinarum*（MG190846.1）（100%）
OV9	MH137696	*Enterobacter ludwigii*（MF101037.1）（100%）
OV16	MH137697	*Enterobacter soli*（MG516168.1）（100%）
OV21	MH137698	*Enterobacter* sp.（KR189383.1）（99%）
OV24	MH137699	*Cedecea* sp.（MG819335.1）（100%）
OV25	MH137700	*Enterococcus gallinarum*（LT223665.1）（99%）
OV28	MH137701	*Lysinibacillus* sp.（MZ604311.1）（100%）
OV32	MH137702	*Enterobacter asburiae*（JQ582973.1）（100%）

Note: OV indicates that bacteria of the adult ovary.

Table S8 Blast-based alignment of 16S rDNA from bacteria in the egg of *P. xylostella*

Clone No.	Accession Number	Blast sequences with highest identity（Accession Number）（%）
RE1	MH141495	E*nterobacter* sp.（KR189383.1）（99%）
RE2	MH141496	*Carnobacterium gallinarum*（MG190846.1）（100%）
RE7	MH141497	*Enterobacter aerogenes*（EU855208.1）（99%）
RE15	MH141498	*Staphylococcus* sp.（MG461552.1）（100%）
RE26	MH141499	*Pseudomonas fulva*（KY511074.1）（100%）
RE41	MH141500	*Enterobacter ludwigii*（MF101037.1）（100%）
SE1	MH141501	*Lysinibacillus macroides*（MG266474.1）（100%）

Note: RE and SE indicate that bacteria of eggs.

Table S9 Different sources of bacteria in Phylum level

Strain	Radish seedlings	Gut			Ovary	Egg
		Larva	Pupa	Adult		
Proteobacteria	√	√	√	√	√	√
Actinobacteria	√	√	√	√		
Bacteroidetes	√					
Firmicutes			√		√	√

Note: √ indicates that bacteria are predasent in the corresponding tissues.

Table S10 Different sources of bacteria in Genus's level

Strain	Radish seedlings	Gut			Ovary	Egg
		Larva	Pupa	Adult		
Enterobacter sp.	√	√	√	√	√	√
Pantoea sp.	√	√			√	
Cedecea sp.	√	√			√	
Pseudomonas sp.	√			√		√
Brachybacterium sp.			√	√		
Enterococcus sp.			√		√	
Carnobacterium sp.					√	√
Lysinibacillus sp.					√	√

Note: √ indicates that bacteria are present in the corresponding tissues.
